# Working in preschool increases the risk of hearing-related symptoms: a cohort study among Swedish women

**DOI:** 10.1007/s00420-019-01453-0

**Published:** 2019-07-08

**Authors:** Sofie Fredriksson, Jeong-Lim Kim, Kjell Torén, Lennart Magnusson, Kim Kähäri, Mia Söderberg, Kerstin Persson Waye

**Affiliations:** 1 Section of Occupational and Environmental Medicine, Dept. of Public Health and Community Medicine, Institute of Medicine, Sahlgrenska Academy, University of Gothenburg, Gothenburg, Sweden; 2 Department of Audiology, Institute of Neuroscience and Physiology, Sahlgrenska Academy, University of Gothenburg, Gothenburg, Sweden; 3 School of Health Sciences, Swedish Institute for Disability Research (SIDR), Örebro University, Örebro, Sweden

**Keywords:** Hyperacusis, Sound-induced auditory fatigue, Tinnitus, Difficulty perceiving speech, Occupational noise, Stressful-working conditions

## Abstract

**Purpose:**

To assess whether working in preschools increases the risk of hearing-related symptoms and whether age, occupational noise, and stressful working conditions affect the risk.

**Methods:**

Questionnaire data on hearing-related symptoms were analysed in women aged 24–65 (4718 preschool teachers, and 4122 randomly selected general population controls). Prevalence and risk ratio (RR) of self-reported hearing loss, tinnitus, difficulty perceiving speech, hyperacusis and sound-induced auditory fatigue were assessed by comparing the cohorts in relation to age and self-reported occupational noise and stressful working conditions (effort–reward imbalance and emotional demands). RR was calculated using log-binomial regression models adjusted for age, education, income, smoking, hearing protection, and leisure noise. Incidence rates and incidence rate ratios (IRR) were calculated for retrospectively reported onset of all symptoms except sound-induced auditory fatigue.

**Results:**

Compared to the controls, preschool teachers had overall more than twofold RR of sound-induced auditory fatigue (RR 2.4, 95% confidence interval 2.2–2.5) and hyperacusis (RR 2.3, 2.1–2.5) and almost twofold for difficulty perceiving speech (RR 1.9, 1.7–2.0). Preschool teachers had a threefold IRR of hyperacusis (IRR 3.1, 2.8–3.4) and twofold for difficulty perceiving speech (IRR 2.4, 2.2–2.6). Significantly although slightly less increased RR and IRR were observed for hearing loss and tinnitus. RR and IRR were generally still increased for preschool teachers when stratified by age and occupational exposure to noise and stress.

**Conclusions:**

This large cohort study showed that working as preschool teacher increases the risk of self-reported hearing-related symptoms, indicating a need of preventative measures.

**Electronic supplementary material:**

The online version of this article (10.1007/s00420-019-01453-0) contains supplementary material, which is available to authorized users.

## Introduction

The majority of research on occupational noise has been performed in traditionally male-dominated and high level-exposure occupations such as industry, mining and construction (Concha-Barrientos et al. 2004; Kurmis and Apps 2007; Lie et al. 2016). Most of these studies have assessed hearing loss measured using pure tone audiometry as the main outcome. Thus, there is a well-recognised causal relationship between occupational noise exposure from machines and tools and the outcome hearing loss (Nelson et al. 2005). In contrast, there is a lack of studies assessing the risks of hearing-related symptoms in traditionally female-dominated occupations, such as preschool teachers. In preschools, the main noise sources are children’s voices, screams and playing activity (Persson Waye et al. 2010; Sjödin et al. 2012; Gerhardsson and Nilsson 2013). The sound environment is highly irregular and intermittent, with equivalent sound levels exceeding 85 dBA in one-minute loggins—up to 100 times per hour (Sjödin et al. 2012). The average sound level indoors in Swedish preschools has been measured close to the lower action level 80 dBA Leq (Persson Waye et al. 2010; Sjödin et al. 2012). The action level is regulated by the Swedish Work Authorities to reduce the risk of noise-induced auditory disorder among employees.

In addition to noise, preschool teachers face several psychosocial stressors at work, such as meeting children’s needs, time pressure and being interrupted (Kelly and Berthelsen 1995). Increased hazard ratios for stress-related disorders have been reported for preschool teachers compared to personnel in non-human service occupations (Wieclaw et al. 2006). Interestingly, a Swedish population-based cross-sectional study has reported an association between occupational stressors and hearing-related symptoms (Hasson et al. 2011). The hypothesis, based mainly on experimental research, is that a stress response may modulate hearing sensitivity on a neuro-endocrine level in two ways. They are (a) through activation of the hypothalamic–pituitary–adrenal axis via glucocorticoid receptors in the cochlea (Canlon et al. 2007), and (b) through sympathetic stimulation affecting cochlear blood flow via adrenergic α-receptors within the cochlea (Bielefeld and Henderson 2007). Prolonged exposure to stress without sufficient recovery is thought to cause an abnormal functioning of the stress response (McEwen 2006), which has been hypothesised to cause auditory disorders (Canlon et al. 2013). However, the causal effect between stress exposure and hearing disorder has not been thoroughly studied in humans.

Non-occupational factors may also be associated to hearing-related symptoms. Previous studies have found associations between smoking and hearing loss (Cruickshanks et al. 1998; Ferrite and Santana 2005), and self-reported hearing problems have been found more common in low socioeconomic groups (Hasson et al. 2010).

A smaller cross-sectional study has indicated that preschool personnel have a high prevalence of hearing-related symptoms: 31% prevalence of tinnitus and 45% prevalence of hyperacusis among 101 personnel surveyed (Sjödin et al. 2012). This is considerably higher than the prevalence found in the general Swedish population, which is about 10–15% for tinnitus and about 8–9% for hyperacusis, depending on symptom definition (Axelsson and Ringdahl 1989; Andersson et al. 2002; Hasson et al. 2010; Paulin et al. 2016). To the best of our knowledge, no previous studies have compared hearing-related symptoms among preschool personnel and randomly selected population controls. Thus, risk estimates of hearing-related symptoms are lacking for this occupational group.

The aim of this study was, therefore, to assess whether working in preschools increases the relative risk of hearing-related symptoms and whether age, exposure to occupational noise or stressful working conditions affect the risk.

## Methods

### Study design and population

**Fig. 1:**
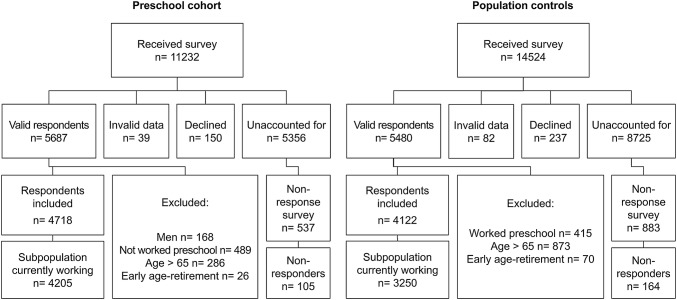
Flow chart of data collection showing initial population, response rates, exclusion criteria and the final study sample of respondents and non-respondents within each of the two cohorts

This cohort study includes baseline and retrospective data collected by postal questionnaires sent out in 2013 and 2014 to 11232 preschool teachers and 14524 women from the general population in Sweden. The preschool cohort included all individuals with a preschool teachers’ degree issued between 1980 and 2012 from universities in the Västra Götaland County of Sweden. The control cohort, which was randomly selected from the Swedish Population and Tax Agency Register, included women born between 1943 and 1989 and currently residing in Västra Götaland County. The response rate to the questionnaire survey was 51% in the preschool cohort and 38% in the population control cohort. As seen in Fig. 1, the final study sample included women 24–65 years of age (born between 1948 and 1989) and consisted of 4718 preschool teachers who had worked in preschools and 4122 controls who, based on an assessment of free text responses on occupational history, had not reported working in preschools. A sub-analysis was also performed including only women currently working, for whom questionnaire data on exposure to occupational noise and stressful working-conditions was available; the sub-analysis included 4205 preschool teachers and 3250 controls.

About 6 months after the first mailing and two reminders were sent, 10% of non-respondents within each cohort were randomly selected for a shorter non-response survey. In total, 105 non-responders within the preschool cohort and 164 within the control cohort returned the non-response questionnaire.

### Outcome and predictor variables

The prevalence of current self-reported symptoms and incidence rates based on retrospectively reported symptom onset were assessed using a questionnaire assessing five hearing-related symptoms as outcomes: self-reported hearing loss, tinnitus, difficulty perceiving speech, hyperacusis and sound-induced auditory fatigue. Self-reported hearing loss was defined by a “yes” response to the question: “Do you have a hearing loss?”. Difficulty perceiving speech was defined by “yes” responses to both work and leisure time for the question: “Do you [at work/in leisure time] have difficulty perceiving speech in an environment where several people are speaking at the same time?”. Tinnitus, hyperacusis and sound-induced auditory fatigue were defined by “sometime each week or more often” responses to the questions: “Do you have tinnitus (a ringing, whizzing or other sound without an external source)?”, “Are you sensitive to sounds (feel discomfort or pain by everyday sounds)?” and “Do you during or after work experience ‘sound fatigue’?”, respectively. Occurrence of symptoms was reported as age and/or year of onset in free text to the question “When did you first notice [the symptom]?”Age of onset was recalculated to calendar year of onset, and onset was excluded if the two measures of occurrence differed > 3 years. The survey did not include onset for sound-induced auditory fatigue. Identical symptom definitions have been validated among women exposed to moderately or high sound levels in obstetrics care (Fredriksson et al. 2016).  In the non-response survey, only self-reporting of hearing loss and tinnitus were included. Tinnitus was assessed using a binary response scale corresponding to the definition of tinnitus detailed above. Self-reported hearing loss was assessed identically as in the larger survey.

Current exposure to occupational noise was assessed with two items: one item reflected whether noise at work was so loud to the extent that conversation was difficult to hear and the other item, whether noise at work was so loud that the person had to raise their voice to communicate. Noise exposure was defined by a report of “about 25% of time” at work or more on one or both of the items. Similar items have been validated in other studies (Neitzel et al. 2009; Schlaefer et al. 2009). Current exposure to stressful working conditions was measured using the effort–reward imbalance (ERI) short version questionnaire, which includes ten items (Siegrist et al. 2004), and the short version of emotional demands from the Copenhagen Psychosocial Questionnaire (COPSOQ), which includes two items assessing experiences of emotionally difficult situations and emotional effects, respectively (Kristensen et al. 2005). For the ERI, one missing value within each dimension was allowed, and replaced by the individual mean from the remaining items in that dimension. A continuous ratio between the effort dimension and the reward dimension was calculated. According to standard praxis, respondents were defined as being exposed to stressful working conditions if they had ERI ratio > 1 (indicating inadequate rewards in relation to the efforts) and/or reported “often” or “always” on both COPSOQ-items. A combined current exposure of noise and stressful working conditions was defined as meeting both the noise and stress definitions, and currently unexposed to noise and stressful working conditions was defined as not meeting any of these definitions of exposure.

We also assessed possible confounding by variables including age, highest attained education level and household income (combined as a proxy for socioeconomic status), smoking (ever smoked daily during at least 1 month), use of hearing protection at work and leisure noise exposure. Leisure noise was assessed as a continuous variable using an index calculated as a sum score from items measuring noisy leisure activities, use of hearing protection in leisure time and listening to music with headphones. A similar index was used in a previous study (Fredriksson et al. 2015). Other potential risk factors for hearing-related disorder, such as a family history of hearing loss, were also collected.

All questionnaire items, response alternatives and variable definitions are presented in Online Resource 1.

### Statistical analyses

Statistical analyses were performed using SAS version 9.4 for Windows (SAS Institute Inc., Cary, NC, USA). Differences in proportions of demographic variables between cohorts were analysed using Chi square test and Mann–Whitney for medians. Point prevalence was calculated as the proportion of cases reporting a currently occurring symptom at the time of survey divided by the total number within each cohort included in the analysis. Log-binomial regression was used to analyse risk ratios (RR) and 95% confidence intervals (CI), with the population control cohort as the reference. RRs were assessed in relation to age categorised into five discrete strata. In the analyses of RR in relation to occupational noise and stressful working conditions, only women currently working were included. Regression models were adjusted for potential confounders including age, education and income, smoking, use of hearing protection at work and leisure noise index. In addition, a test of trend (Mantel–Haenszel Chi square test) was used to assess whether symptom prevalence increased by age category within each cohort, and by exposure strata within each cohort. The order of the exposure categories was assumed as: unexposed followed by stress only, noise only, and lastly, both noise and stress). Incidence rates (IR) were calculated as retrospectively reported symptom onset between age 24 and 65 divided by the sum of person-years at risk (number of years after age 24) between the ages of 24–65, and presented per 1000 person-years. For IR, the 95% CI was calculated as approximating a Poisson distribution (Rosner 2015). Incidence rate ratios (IRR) were calculated with the test-based 95% CI (Miettinen 1976). The population control cohort was used as the reference. The recall bias of retrospectively reported symptom onset was assessed visually in Kaplan–Meier survival curves stratified by event recall time (reporting the onset as occurring 5, 10, or 15 years prior to the survey). Finally, a non-response analysis of RR for self-reported hearing loss and tinnitus was assessed by comparing non-respondents to respondents separately within each cohort, using log-binomial regression and adjusting for age, with respondents as the reference. In the non-response analysis, sampling weights were used and standard errors were corrected for the difference in sample size between respondents and non-respondents (SAS usage note 23003, available at http://support.sas.com/kb/23/003.html, retrieved 16-07-07). The significance level was set at 5% (*p *= 0.05) for all tests.

## Results

**Fig. 2:**
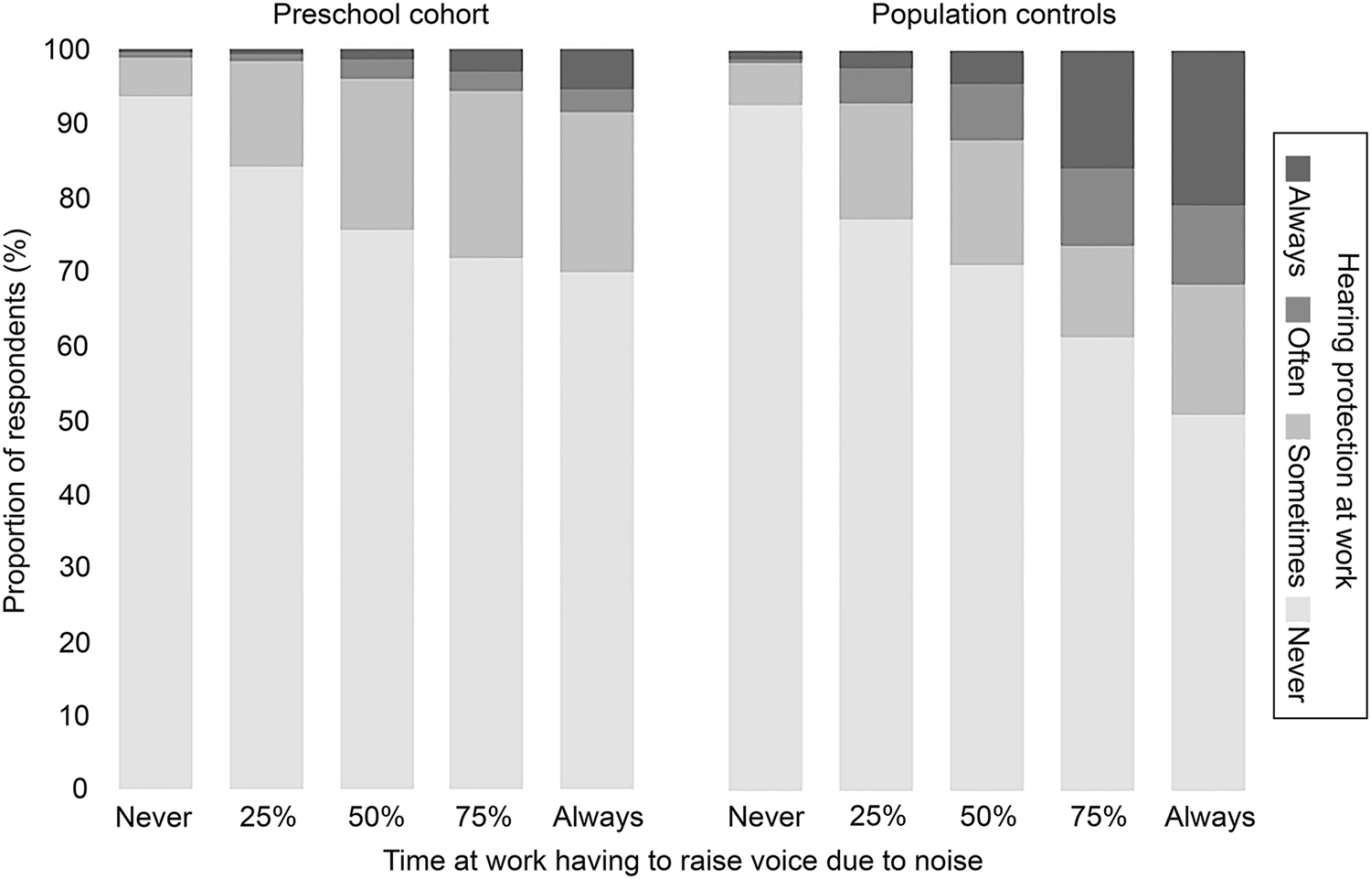
Use of hearing protection at work (frequency of use shown in different shades of grey) in relation to noise exposure at work (*x*-axis, time at work having to raise voice due to noise) shown as proportion of respondents (*y*-axis) in each noise exposure strata for the preschool cohort (left) and for population controls (right)

A major difference between the two cohorts was found in the proportion reporting occupational noise exposure. As shown in Table 1, 75% of the preschool teachers reported having to raise their voice due to noise at work, compared to 29% among the controls. Despite this, fewer preschool teachers reported use of hearing protection at work (Fig. 2). Preschool teachers also reported stressful working conditions more frequently than controls. For example, effort–reward imbalance was found in 80% of the preschool cohort, compared to 59% in the control cohort. Due to the difference in selection criteria for the two cohorts, there was also a large difference in education level (Table 1). Median years of working in preschool amongst the preschool teachers was 12 years (IQR 6–20 years), based on data from *n* = 4566 preschool teachers. Despite our efforts of excluding controls who had worked in preschool, a post hoc analysis showed that a very small proportion (0.2%, *n* = 94) of the control cohort had worked as child caretaker, most likely in preschool, with a median of 5.5 working years (IQR 2–13 years).

**Table 1:** Demographic data on female preschool teachers and randomly selected women as population controls

	Preschool cohort (total *n* = 4718)^a^				Population controls (total *n* = 4122)^a^				*p* value^b^
	Median (IQR)	*n*/total *n*	%	(95% CI)	Median (IQR)	*n*/total *n*	%	(95% CI)	
Age in years	45 (38–53)				48 (39–57)				< 0.0001
Employment status (currently working)		4265/4714	90	(90–91)		3310/4114	80	(79–82)	< 0.0001
Education and income combined (mutually exclusive categories)^c^									< 0.0001
University education and ≥ 30,000 SEK		3804/4653	82	(81–83)		1774/4027	44	(43–46)	
No university education and ≥ 30,000 SEK, or, university education and < 30,000 SEK		849/4653	18	(17–19)		1528/4027	38	(36–39)	
Lower than university education and < 30,000 SEK		0/4653	0	–		725/4027	18	(17–19)	
Smoking (ever smoked daily)		1213/4703	26	(25–27)		1591/4089	39	(37–40)	< 0.0001
Family history of hearing loss (< age 55)		881/4702	19	(18–20)		755/4100	18	(17–20)	0.698
Ear infections (recurrent or prolonged)		728/4690	16	(14–17)		580/4092	14	(13–15)	0.077
Tympanostomy tube (ever)		191/3351	6	(5–6)		149/2743	5	(5–6)	0.651
Noisy leisure activities (≥ month or more)		1179/4705	25	(24–26)		1199/4089	29	(28–31)	< 0.0001
Hearing protection leisure time (always or often)^d^		208/1164	18	(16–20)		233/1182	20	(17–22)	0.253
Loud music in headphones (≥ month, ≥ 75% vol.)		358/2695	13	(12–15)		460/2458	19	(17–20)	< 0.0001
Loud noise, can´t hear conversation (≥ 25% time)		3368/4515	75	(73–76)		1176/3688	32	(30–33)	< 0.0001
Loud noise, have to raise own voice (≥ 25% time)		3376/4517	75	(73–76)		1078/3689	29	(28–31)	< 0.0001
Hearing protection at work (always or often)		123/4521	3	(2–3)		170/3698	5	(4–5)	< 0.0001
Changed job/workplace due to noise (ever)		312/4685	7	(6–7)		72/4034	2	(1–2)	< 0.0001
*Stressful working conditions*									
Effort–reward imbalance (ERI) (ratio > 1)		3725/4684	80	(78–81)		2383/4012	59	(58–61)	< 0.0001
Emotional demands, COPSOQ (often or always)		1699/4663	36	(35–38)		919/3985	23	(22–24)	< 0.0001
Exposure strata among currently working^e^									< 0.0001
Unexposed to noise and stress		384/4205	9	(8–10)		919/3250	29	(27–30)	
Stress only (ERI or COPSOQ)		831/4205	20	(19–21)		1483/3250	46	(44–48)	
Noise only (exposed ≥ 25% of time at work)		294/4205	7	(6–8)		163/3250	5	(4–6)	
Both noise and stress exposure		2696/4205	64	(63–66)		685/3250	21	(20–22)	

*IQR* inter-quartile range

^a^Table shows data for subjects with non-missing data

^b^*p* values based on non-parametric test of difference in medians or Chi square test of difference in proportions between the two cohorts

^c^All preschool teachers have a university degree and data was obtained from national registry. For controls, the proportion reporting university as the highest attained education level are shown. The rest had compulsory schooling or lower

^d^Proportion of non-missing data among those reporting noisy leisure activities

^e^Exclusive categories among currently working respondents excluding individuals with missing data on noise and/or stress exposure

The main result was the significantly increased risk of hearing-related symptoms among preschool teachers compared to controls. As seen in Table 2, the overall adjusted RR was more than twofold for sound-induced auditory fatigue and for hyperacusis, and almost twofold for difficulty perceiving speech. For the self-reported symptoms hearing loss and tinnitus, the risk estimates were also significantly increased although to a lesser degree. When stratifying by age, adjusted RRs were still increased for the preschool cohort and significantly so in all, but the youngest age strata for hearing loss and tinnitus (Table 2). Symptom prevalence was generally increased with increased age in both cohorts (*p *< 0.05), with the exception of hyperacusis and sound-induced auditory fatigue. Prevalence of hyperacusis did not increase by age among the controls (*p *=0.971), and sound-induced auditory fatigue did not increase by age among preschool teachers (*p *= 0.551), nor among controls (*p *= 0.644).

**Table 2:** 2013–2014 year prevalence of hearing-related symptoms and risk ratios, among female preschool teachers compared to randomly selected women as population controls, in relation to age

	Prevalence (%)						Risk ratio (RR)^a^ (preschool/control)			
	Preschool cohort (total *n* = 4718)^b^			Population controls (total *n* = 4122)^b^			Crude		Adjusted^c^	
	*n*/total *n*	%	95% CI	*n*/total *n*	%	95% CI	RR	95% CI	RR	95% CI
*Hearing loss*										
24–29	24/286	8	(5–12)	26/384	7	(4–9)	1.2	(0.7–2.1)	1.4	(0.8–2.5)
30–39	119/1114	11	(9–13)	57/725	8	(6–10)	**1.4**	(1.0–1.8)	**1.7**	(1.2–2.4)
40–49	269/1579	17	(15–19)	122/1048	12	(10–14)	**1.5**	(1.2–1.8)	**1.7**	(1.4–2.1)
50–59	356/1398	25	(23–28)	211/1147	18	(16–21)	**1.4**	(1.2–1.6)	**1.6**	(1.3–1.9)
60–65	101/293	34	(29–40)	172/749	23	(20–26)	**1.5**	(1.2–1.8)	**1.8**	(1.4–2.3)
All	869/4670	19	(17–20)	588/4053	15	(13–16)	**1.3**	(1.2–1.4)	**1.6**	(1.5–1.8)
*Tinnitus*										
24–29	30/288	10	(7–14)	48/387	12	(9–16)	0.8	(0.6–1.3)	1.0	(0.6–1.8)
30–39	145/1122	13	(11–15)	60/726	8	(6–10)	**1.6**	(1.2–2.1)	**1.6**	(1.2–2.2)
40–49	273/1580	17	(15–19)	114/1052	11	(9–13)	**1.6**	(1.3–2.0)	**1.9**	(1.5–2.4)
50–59	332/1397	24	(22–26)	183/1155	16	(14–18)	**1.5**	(1.3–1.8)	**1.6**	(1.4–2.0)
60–65	78/297	26	(21–31)	150/753	20	(17–23)	**1.3**	(1.0–1.7)	**1.8**	(1.4–2.5)
All	858/4684	18	(17–19)	555/4073	14	(13–15)	**1.3**	(1.2–1.5)	**1.7**	(1.5–1.9)
*Difficulty perceiving speech*										
24–29	94/285	33	(28–38)	72/375	19	(15–23)	**1.7**	(1.3–2.2)	**1.8**	(1.4–2.5)
30–39	429/1108	39	(36–42)	145/717	20	(17–23)	**1.9**	(1.6–2.3)	**2.0**	(1.7–2.4)
40–49	745/1576	47	(45–50)	253/1040	24	(22–27)	**1.9**	(1.7–2.2)	**2.0**	(1.8–2.3)
50–59	732/1380	53	(50–56)	354/1137	31	(28–34)	**1.7**	(1.5–1.9)	**1.7**	(1.5–1.9)
60–65	144/280	49	(43–54)	229/713	32	(29–36)	**1.5**	(1.3–1.8)	**1.6**	(1.3–1.9)
All	2136/4629	46	(45–48)	1053/3982	26	(25–28)	**1.7**	(1.6–1.9)	**1.9**	(1.7–2.0)
*Hyperacusis*										
24–29	77/287	27	(22–32)	62/386	16	(12–20)	**1.7**	(1.2–2.3)	**1.7**	(1.2–2.4)
30–39	420/1121	37	(35–40)	144/730	20	(17–23)	**1.9**	(1.6–2.2)	**2.0**	(1.7–2.5)
40–49	649/1585	41	(39–43)	198/1053	19	(16–21)	**2.2**	(1.9–2.5)	**2.3**	(2.0–2.7)
50–59	582/1402	42	(39–44)	213/1162	18	(16–21)	**2.3**	(2.0–2.6)	**2.5**	(2.1–2.9)
60–65	102/299	34	(29–39)	134/752	18	(15–21)	**1.9**	(1.5–2.4)	**2.3**	(1.7–3.1)
All	1830/4694	39	(38–40)	751/4083	18	(17–20)	**2.1**	(2.0–2.3)	**2.3**	(2.1–2.5)
*Sound-induced auditory fatigue*										
24–29	205/288	71	(66–76)	119/385	31	(26–36)	**2.3**	(2.0–2.7)	**1.2**	(1.1–1.3)
30–39	805/1126	71	(69–74)	232/724	32	(29–35)	**2.2**	(2.0–2.5)	**2.4**	(2.1–2.7)
40–49	1140/1585	72	(70–74)	340/1047	32	(30–35)	**2.2**	(2.0–2.4)	**2.3**	(2.1–2.5)
50–59	1002/1403	71	(69–74)	373/1155	32	(30–35)	**2.2**	(2.0–2.4)	**1.5**	(1.4–1.6)
60–65	201/295	68	(63–73)	239/735	33	(29–36)	**2.1**	(1.8–2.4)	**2.2**	(1.9–2.6)
All	3353/4697	71	(70–73)	1303/4046	32	(31–34)	**2.2**	(2.1–2.3)	**2.4**	(2.2–2.5)

^a^Bold indicates significant risk ratio estimate from log-binomial regression (*p* < 0.05)

^b^Table shows data for subjects with non-missing data

^c^Adjusted for age (except for the age-stratified RR), education and income combined, smoking, hearing protection at work and leisure noise index

In addition, the exposure-stratified analysis, performed among women currently working, showed that preschool teachers still had increased adjusted RRs of hearing-related symptoms compared to controls when taking current exposure to occupational noise and stressful working conditions into account (Table 3). The increased risks observed for preschool teachers were statistically significant in most exposure strata, but also within the unexposed strata. The prevalence of hearing-related symptoms was most pronounced within the strata exposed to both noise and stressful working conditions for both cohorts, and the test of trend showed a significant increase in prevalence from the unexposed category through to exposure to both noise and stressful working conditions for all symptoms in both cohorts (*p *< 0.05).

**Table 3:** 2013–2014 year prevalence of hearing-related symptoms and risk ratios, among currently working female preschool teachers compared to currently working randomly selected women as population controls, in relation to occupational exposure

	Prevalence (%)						Risk ratio (RR)^b^ (preschool/control)			
	Preschool cohort (total *n* = 4205)^a^			Population controls (total *n* = 3250)^a^			Crude		Adjusted^c^	
	*n*/total *n*	%	(95% CI)	*n*/total *n*	%	(95% CI)	RR	(95% CI)	RR	(95% CI)
*Hearing loss*										
Unexposed to noise and stress	55/378	15	(11–18)	96/913	11	(9–13)	**1.4**	(1.0–1.9)	**1.6**	(1.1–2.2)
Stress only	106/825	13	(11–15)	188/1469	13	(11–15)	1.0	(0.8–1.3)	1.2	(0.97–1.6)
Noise only	60/293	20	(16–25)	30/160	19	(13–25)	1.1	(0.7–1.6)	1.3	(0.8–2.2)
Both noise and stress	566/2676	21	(20–23)	131/673	19	(16–22)	1.1	(0.9–1.3)	**1.4**	(1.1–1.7)
All	787/4172	19	(18–20)	445/3215	14	(13–15)	**1.4**	(1.2–1.5)	**1.7**	(1.5–1.9)
*Tinnitus*										
Unexposed to noise and stress	51/382	13	(10–17)	76/915	8	(7–10)	**1.6**	(1.2–2.2)	**2.1**	(1.4–3.0)
Stress only	104/827	13	(10–15)	180/1476	12	(11–14)	1.0	(0.8–1.3)	1.2	(0.9–1.5)
Noise only	48/294	16	(12–21)	17/161	11	(6–15)	1.5	(0.9–2.6)	1.6	(0.8–2.9)
Both noise and stress	560/2679	21	(19–22)	130/681	19	(16–22)	1.1	(0.9–1.3)	**1.4**	(1.2–1.7)
All	763/4182	18	(17–19)	403/3233	12	(11–14)	**1.5**	(1.3–1.6)	**1.8**	(1.6–2.0)
*Difficulty perceiving speech*										
Unexposed to noise and stress	101/380	27	(22–31)	155/917	17	(14–19)	**1.6**	(1.3–2.0)	**1.6**	(1.3–2.0)
Stress only	279/825	34	(31–37)	368/1480	25	(23–27)	**1.4**	(1.2–1.5)	**1.5**	(1.3–1.7)
Noise only	128/293	44	(38–49)	46/162	28	(21–35)	**1.5**	(1.2–2.0)	**1.4**	(1.0–2.0)
Both noise and stress	1453/2675	54	(53–56)	312/682	46	(42–49)	**1.2**	(1.1–1.3)	**1.3**	(1.1–1.4)
All	1961/4173	47	(46–49)	881/3241	27	(26–29)	**1.7**	(1.6–1.8)	**1.9**	(1.7–2.0)
*Hyperacusis*										
Unexposed to noise and stress	67/383	17	(14–21)	93/915	10	(8–12)	**1.7**	(1.3–2.3)	**1.7**	(1.2–2.3)
Stress only	191/830	23	(20–26)	230/1477	16	(14–17)	**1.5**	(1.2–1.8)	**1.5**	(1.2–1.8)
Noise only	76/292	26	(21–31)	26/162	16	(10–22)	**1.6**	(1.1–2.4)	1.3	(0.8–2.1)
Both noise and stress	1305/2685	49	(47–50)	191/681	28	(25–31)	**1.7**	(1.5–2.0)	**1.8**	(1.6–2.1)
All	1639/4190	39	(38–41)	540/3235	17	(15–18)	**2.3**	(2.2–2.6)	**2.4**	(2.2–2.6)
*Sound-induced auditory fatigue*										
Unexposed to noise and stress	98/384	26	(21–30)	88/915	10	(8–12)	**2.7**	(2.0–3.4)	**2.7**	(2.0–3.6)
Stress only	370/828	45	(41–48)	368/1477	25	(23–27)	**1.8**	(1.6–2.0)	**1.6**	(1.5–1.8)
Noise only	192/292	66	(60–71)	59/163	36	(29–44)	**1.8**	(1.5–2.3)	**1.5**	(1.2–2.0)
Both noise and stress	2353/2690	87	(86–89)	463/683	68	(64–71)	**1.3**	(1.2–1.4)	**1.3**	(1.2–1.4)
All	3013/4194	72	(70–73)	978/3238	30	(29–32)	**2.4**	(2.3–2.5)	**1.9**	(1.8–1.9)

^a^Including only women currently working with data on occupational exposures. Table shows data for subjects with non-missing data

^b^Bold indicates significant risk ratio estimate from log-binomial regression (*p* < 0.05)

^c^Adjusted for age, education and income combined, smoking, hearing protection at work and leisure noise index

Furthermore, the preschool cohort reported the first occurrence of symptoms (symptom onset) earlier in life compared to the controls. Thus, incidence rates were higher in the preschool cohort than in the control cohort resulting in significantly increased IRRs for the preschool cohort compared to the control cohort for all the symptoms assessed (Table 4). Hyperacusis and difficulty perceiving speech displayed a tripled and doubled IRR, respectively, for the preschool cohort. The effect of recall due to retrospective report of symptom onset was similar in both cohorts, as shown by generally parallel Kaplan–Meier survival curves stratified by different length of recall time (Online Resource 2).

**Table 4:** Incidence rates and incidence rate ratio of hearing-related symptoms with onset between age 24–65, among female preschool teachers compared to randomly selected women as population controls, in relation to birth year ^a^

	Incidence rate (IR) (cases/1000 person-years)						Incidence rate ratio (IRR)^b^ (preschool/control)	
	Preschool cohort (total *n* = 4718)^c^			Population controls (total *n* = 4122)^c^			Crude	
	*n*/total *n*	IR	(95% CI)	*n*/total *n*	IR	(95% CI)	IRR	(95% CI)
*Hearing loss*								
Birth cohort 1989–1984	9/271	10.1	(3.5–16.7)	3/361	2.9	(− 0.4–6.3)	**3.4**	(1.0–11.7)
Birth cohort 1984–1974	67/1062	6.0	(4.5–7.4)	19/687	2.6	(1.4–3.8)	**2.3**	(1.4–3.7)
Birth cohort 1974–1964	210/1520	7.2	(6.2–8.2)	71/997	3.5	(2.7–4.4)	**2.0**	(1.6–2.6)
Birth cohort 1964–1954	284/1326	7.7	(6.8–8.6)	152/1088	4.8	(4.1–5.6)	**1.6**	(1.3–1.9)
Birth cohort 1954–1948	87/279	9.2	(7.3–11.2)	138/715	5.3	(4.5–6.2)	**1.7**	(1.3–2.3)
All	657/4458	7.5	(6.9–8.1)	383/3848	4.5	(4.0–4.9)	**1.7**	(1.5–1.9)
*Tinnitus*								
Birth cohort 1989–1984	17/275	19.1	(10.0–28.2)	9/348	9.0	(3.1–14.9)	**2.1**	(1.0–4.7)
Birth cohort 1984–1974	109/1086	9.6	(7.8–11.4)	30/696	4.1	(2.6–5.6)	**2.3**	(1.6–3.5)
Birth cohort 1974–1964	251/1558	8.4	(7.4–9.5)	87/1025	4.2	(3.3–5.1)	**2.0**	(1.7–2.5)
Birth cohort 1964–1954	311/1376	8.0	(7.1–8.9)	159/1131	4.8	(4.1–5.6)	**1.7**	(1.4–2.0)
Birth cohort 1954–1948	73/292	7.1	(5.6–8.7)	134/737	5.0	(4.1–5.8)	**1.4**	(1.1–1.9)
All	761/4587	8.4	(7.8–9.0)	419/3937	4.7	(4.3–5.2)	**1.8**	(1.6–2.0)
*Difficulty perceiving speech*								
Birth cohort 1989–1984	58/249	77.7	(57.7–97.8)	24/327	26.5	(15.9–37.0)	**2.9**	(1.9–4.6)
Birth cohort 1984–1974	347/1026	36.2	(32.4–40.0)	92/664	13.8	(11.0–16.7)	**2.6**	(2.1–3.3)
Birth cohort 1974–1964	676/1507	25.8	(23.9–27.8)	193/980	10.3	(8.8–11.7)	**2.5**	(2.2–2.9)
Birth cohort 1964–1954	675/1323	19.8	(18.3–21.2)	301/1084	10.0	(8.8–11.1)	**2.0**	(1.7–2.3)
Birth cohort 1954–1948	121/265	13.7	(11.3–16.1)	198/682	8.2	(7.0–9.3)	**1.7**	(1.3–2.1)
All	1877/4370	23.6	(22.5–24.7)	808/3737	10.0	(9.3–10.7)	**2.4**	(2.2–2.6)
*Hyperacusis*								
Birth cohort 1989–1984	56/266	69.7	(51.5–88.0)	10/334	10.6	(4.0–17.1)	**6.6**	(3.7–11.8)
Birth cohort 1984–1974	350/1051	35.2	(31.5–38.9)	90/676	13.3	(10.5–16.0)	**2.7**	(2.1–3.3)
Birth cohort 1974–1964	586/1522	21.9	(20.1–23.6)	146/1001	7.4	(6.2–8.6)	**2.9**	(2.5–3.5)
Birth cohort 1964–1954	530/1350	14.8	(13.6–16.1)	179/1128	5.5	(4.7–6.3)	**2.7**	(2.3–3.2)
Birth cohort 1954–1948	92/289	9.2	(7.4–11.1)	114/732	4.3	(3.5–5.0)	**2.2**	(1.7–2.8)
All	1614/4478	19.4	(18.5–20.3)	539/3871	6.2	(5.7–6.8)	**3.1**	(2.8–3.4)
*Sound-induced auditory fatigue* ^d^		N/A			N/A			

^a^Subjects in birth cohort 1989–1984 had age 24–29 at year of survey (2013–2014), birth cohort 1984–1974 had age 30–39, birth cohort 1974–1964 had age 40–49, birth cohort 1964–1954 had age 50–59 and birth cohort 1954–1948 had age 60–65. Hence, strata are mutually exclusive categories

^b^Bold indicates significant incidence rate ratio estimate from log-binomial regression (*p* < 0.05)

^c^Table shows data for subjects with non-missing data

^d^Data on incidence (symptom onset year) for sound-induced auditory fatigue was not collected

Lastly, the non-response analysis showed that, compared to responders, non-responders in both cohorts had increased age-adjusted RR of self-reported hearing loss (preschool cohort RR: 1.4, 95% CI 1.3–1.6; control cohort RR: 1.4, 95% CI 1.2–1.5). For tinnitus, RR was significantly increased for non-responders in the preschool cohort (RR: 1.2, 95% CI 1.1–1.3), but not in the control cohort (RR 0.9, 95% CI 0.8–1.0).

## Discussion

The main finding of this study was the increased risks of self-reported hearing-related symptoms among female preschool teachers, compared to women in the general population. To the best of our knowledge, the current study is the first to present relative risk estimates for this occupational group.

Although causal effects of occupational exposure to noise and stress were not explicitly assessed, we found support for the importance of these occupational factors for hearing-related symptoms. Firstly, a larger proportion of preschool teachers reported these occupational exposures compared to controls. These results are in line with previous studies showing that preschool teachers are exposed to high sound levels in preschool (Persson Waye et al. 2010; Sjödin et al. 2012; Gerhardsson and Nilsson 2013) and have an increased risk of stressful working conditions compared to non-human service occupations (Wieclaw et al. 2006). In addition, other potential risk factors, such as smoking and low socioeconomic status were less common among preschool teachers. Nonetheless, we cannot neglect the possibility that these factors confound our main findings. However, when adjusting for smoking, we still observed the same findings with only minor changes in the risk estimates. Secondly, a high symptom prevalence was found among those currently exposed to noise and stress at work compared to those who were defined as unexposed. Thirdly, we found that preschool teachers seldom use hearing protection at work even though a majority of them report being exposed to high sound levels at work. This could further explain the increased risk, as preschool teachers would per se be more exposed than controls who report similar extent of noise exposure. A similar behaviour has been reported among musicians (Laitinen 2005). Taken together, these results strongly indicate an importance of the preschool work environment in explaining the identified increase in risk of hearing-related symptoms among preschool teachers.

Researchers have argued that a functioning stress response is vital for the auditory system (Hasson et al. 2013). Although stressful working conditions is an important factor within human service professions such as preschool teaching (Wieclaw et al. 2006), whether it has a causal effect on hearing-related symptoms needs to be studied further. Interestingly, the increase in prevalence of hearing-related symptoms observed in the combined noise and stress exposure strata in the current study indicate a possible interaction effect between noise and stress. A previous study has showed an additive effect of exposure to both stressful working conditions, assessed as job-strain, and road-traffic noise on myocardial infarction (Selander et al. 2013). It should be noted that the Job-Demand-Control, used to assess job-strain in the cited study, to a larger extent focus on instrumental contents compared to the ERI model used in the current study, which emphasise social aspects of work.

In the current study, one of the most notable increases in risk was seen for the symptom hyperacusis, with more than a twofold RR and as much as a threefold IRR for the preschool teachers compared to the controls. Although different aetiologies involving both peripheral and central underlying factors may explain hyperacusis, noise exposure has been suggested to be one of the most common causes, and noise has been used in experimental studies to induce hyperacusis (Aazh et al. 2014; Pienkowski et al. 2014). Hyperacusis has previously been found prevalent among rock and jazz musicians who are exposed to high sound levels (Kähäri et al. 2003), also indicating an effect of noise exposure. In addition, studies have shown that teachers and childcare professionals are common occupations among hyperacusis patients together with musicians (Anari et al. 1999; Jüris et al. 2013). The prevalence of hyperacusis among preschool teachers in this study is similar to a previous study reporting that 45% out of 101 Swedish preschool personnel experienced hyperacusis “sometimes” or “quite often” (Sjödin et al. 2012). Hyperacusis has been defined as “discomfort for sounds that would be acceptable to most people” (Khalfa et al. 2002), where moderately intense sounds are judged as very loud (Tyler et al. 2014), or even painful (Baguley 2014). As hyperacusis can lead to avoidance and fear of everyday sounds (Bläsing et al. 2010; Blaesing and Kroener-Herwig 2012), the unpredictable and irregular sound environment in preschools can be expected to be particularly disabling for preschool personnel with hyperacusis. These consequences might influence the prevalence and could possibly further explain the pronounced increase in risk among the preschool teachers compared to the controls.

The risk of self-reported hearing loss was less pronounced compared to other symptoms in our study. A review recently concluded that there is insufficient evidence for occupational noise in preschool causing elevated pure tone hearing thresholds and that noise levels are probably too low to cause such hearing loss (Lie et al. 2016). Preschool personnel have been reported to have slightly worse pure tone hearing thresholds compared to reference data, although most had thresholds within the “normal range”, i.e., better than 20–25 dB HL (Sjödin et al. 2012). It is worth noting though that elevated pure tone thresholds even within the normal range may be indicative of disorder. It is possible that self-reporting of hearing loss cannot fully capture slightly elevated pure tone thresholds. This is in line with previous validation studies indicating a higher sensitivity for self-reported hearing loss in relation to more severely elevated pure tone thresholds (Nondahl et al. 1998; Sindhusake et al. 2001; Fredriksson et al. 2016). Based on earlier research among preschool teachers, we had expected that tinnitus would be more common in the preschool cohort (Sjödin et al. 2012). Instead, occurrence and relative risk of tinnitus were less pronounced compared to the other symptoms. The prevalence estimates among controls were, however, comparable to that of earlier population studies (Axelsson and Ringdahl 1989; Hasson et al. 2010).

Notably, sound-induced auditory fatigue was clearly the most common symptom in our study. Unlike the other symptoms, the prevalence of sound-induced auditory fatigue was not affected by age, but was particularly high in relation to noise exposure at the current workplace. Unfortunately, the IR of sound-induced auditory fatigue was not available to assess. There is limited previous research on sound-induced auditory fatigue, but we hypothesise that the information content of the noise and the difficulty to wear hearing protection in preschool, and likely also exposure to psychosocial stress, are important factors explaining this symptom. These factors are more prominent in the preschool work environment, which may explain the increased risk among the preschool teachers. Our current results are in line with a previous study in which we found increased odds ratios of sound-induced auditory fatigue among obstetrical personnel in relation to self-reported occupational noise exposure, noise annoyance, and to some degree work-related stress, but not in relation to age (Fredriksson et al. 2015).

The current study has both limitations and strengths. A major strength is the large sample size, which yielded power to detect effects, except in parts of the stratified analyses. The overall response rate to the survey was 38% in the control cohort and 51% in the preschool cohort, and generally lower in the younger age groups compared to the older age groups, particularly among controls. This could affect the generalisability of our results, potentially causing a response bias. However, the non-response analysis indicated relatively minor influence on our main findings and, if anything, a possible underestimation of the risk among responders compared to non-responders. Another limitation is the cross-sectional data. However, collecting retrospective reports of symptom onset gave us the opportunity to also assess incidence. A similar retrospective self-report method has been used successfully for assessing asthma onset (Torén et al. 2006). We found that the effect of recall of retrospectively reported onset was similar in both cohorts, and thus should not bias these risk estimates. Nevertheless, as subjects in the two cohorts may have different demands on hearing ability and awareness of changes in their hearing, it is possible that recall of symptoms onset, influenced by perception bias, will differ slightly in the two cohorts.

The study has limitations regarding interpretation of exposure effects and causal effects, as we focused on comparing two cohorts having a different occupational history and occupational exposure, rather than assessing exposure effects directly. Misclassification of exposure may have affected the results of the exposure-stratified analysis, as we saw increased risk in the unexposed strata and as we only had current exposure available. Further analysis of the direct effects of exposure as well as long-term causal effects in longitudinal studies are needed. Potential misclassification of outcomes due to self-report also limits the extent to which conclusion can be drawn regarding manifestation of specific auditory physiological disorders. To date, symptoms such as tinnitus and hyperacusis, however, are mainly diagnosed by self-report in the clinic.

Future research should also study the consequences of suffering from hearing-related symptoms such as hyperacusis in relation to the ability to continue working in preschool. These consequences may to some extent explain why preschool teachers in this study reported to have changed job due to noise at work to a greater extent than the control cohort has. This also indicates a need for preventative and remedying measures to be taken in the preschool work environment.

## Conclusion

This study showed that working as a preschool teacher significantly increases the relative risk of self-reported hearing-related symptoms compared to women in the general population. The relative risk was generally increased both when stratifying by age and by current exposure to occupational noise and stressful working conditions. Overall, the risk was most pronounced for the symptoms sound-induced auditory fatigue, hyperacusis and difficulty perceiving speech, and increased to a somewhat lesser degree for self-reported hearing loss and tinnitus.

Longitudinal studies are needed to ascertain causal effects and to disentangle the mechanisms for the different symptoms. At present, the high symptom prevalence found in this study among preschool teachers and women exposed to noise and stress at work indicate that these occupational factors are of importance. Thus, preventative measures in the work environment should not be delayed.

## Electronic supplementary material

Below is the link to the electronic supplementary material.

**Online Resource 1** details all questionnaire items, response alternatives and variable definitions (PDF 72 kb)

**Online Resource 2** shows the effect of recall due to retrospective reported symptom onset in Kaplan–Meier survival curves (PDF 364 kb)
