# Supplementary material for: Working in preschool increases the risk of hearing-related symptoms: a cohort study among Swedish women
Source: Int Arch Occup Environ Health. 2019 Jul 8;92(8):1179–90. doi: 10.1007/s00420-019-01453-0 (PMC6814644; doi:10.1007/s00420-019-01453-0)
Supplement: Supplementary file 1 — Online Resource 1 details all questionnaire items, response alternatives and variable definitions (PDF 72 kb) [file 420_2019_1453_MOESM1_ESM.pdf]

Working in preschool increases the risk of hearing-related symptoms - a cohort study among Swedish women, International Archives of Occupational and Environmental Health, Sofie Fredriksson, Jeong-Lim Kim, Kjell Torén, Lennart Magnusson, Kim Kähäri, Mia Söderberg, Kerstin Persson Waye; Department of Occupational and Environmental Medicine, Public Health & Community Medicine, Institute of Medicine, Sahlgrenska Academy, University of Gothenburg, sofie.fredriksson@gu.se

Online Resource 1 Questionnaire items, response alternatives and derived variables. The translation from Swedish has not been validated.

Questionnaire item in full text	Response alternatives	Derived variables
Hearing loss<br>"Do you have a hearing loss?"	- Yes<br>- No<br>- Don't know	Self-reported hearing loss was defined as responding: "yes".<br>Also used in non-response survey.
Difficulty perceiving speech<br>"Do you <u>at work</u> have trouble hearing what is said in an environment where several people are talking at the same time?"<br><br>"Do you <u>in leisure time</u> have trouble hearing what is said in an environment where several people are talking at the same time?"	- Yes<br>- No<br><br>*Same response scale for both items.*	Difficulty perceiving speech was defined as responding: "yes" to both work and leisure time.
Tinnitus<br>"Do you have tinnitus (a ringing, whistling or other sound without an external source), that lasts more than five minutes each time?"	- No<br>- No, but I have had it<br>- Yes, a few times each month or more seldom<br>- Yes, once or twice a week<br>- Yes, many times each week<br>- Yes, everyday	Tinnitus was defined as responding: "Yes, once or twice a week" or more often<br>Also used in non-response survey, where the response scale was:<br>- No<br>- Yes, once or twice a week or more often
Hyperacusis<br>"Are you sensitive to sounds (feel discomfort or pain by everyday sounds)?"	- No<br>- No, but I have had<br>- Yes, a few times each month or more seldom<br>- Yes, once or twice a week Yes, many times each week<br>- Yes, everyday	Hyperacusis was defined as responding: "Yes, once or twice a week" or more often.
Sound-induced auditory fatigue<br>"Do you, during or after work, experience any of the following symptoms or disorders?<br>- Sound fatigue"	- Never, seldom<br>- A few times each month<br>- Once or twice a week<br>- Many times each week<br>- Everyday	Sound-induced auditory fatigue was defined as responding: "once or twice a week" or more often.<br><br>The item was included in a matrix with other symptoms, such as headache and tiredness.
Symptom onset<br>"When did you first notice that you had [hearing loss/ difficulty perceiving speech/tinnitus/hyperacusis]? Try to respond even if you do not remember the exact year or age when it was first noted."	- Year (e.g. 2004)<br>- Age (e.g. 46)<br><br>(free text)	Onset age was used if reported. Onset year was recalculated to onset age if reported. Age was used if both were reported, unless they differed by >3 years. If so, they were treated as missing.
Can't hear conversation due to noise<br>"Is the sound level at your workplace sometimes so high that you have difficulty hearing what other people are saying?"<br><br>Have to raise voice due to noise<br>"How often are you at your workplace exposed to such high sound levels that you have to raise your voice to be able to talk to other people?"	- Never/almost never<br>- About 25% of time<br>- About 50% of time<br>- About 75% of time<br>- Always/almost always<br><br>*Same response scale for both items.*	Reporting "About 25% of time" or more was used as cut-off for each item.<br><br>Noise exposed strata was defined as reporting: "25% of time" or more often on both items.
Hearing protection at work<br>"Do you use earplugs or other hearing protection devices at your current workplace?"	- Always/almost always<br>- Often<br>- Sometimes<br>- Never/rarely	Hearing protection at work defined as responding: "always" or "often".
Changed job or workplace due to noise<br>"Have you ever changed job or workplace due to noise or high sound levels?"	- No<br>- Yes	Having ever changed job due to noise was defined as responding: "yes".
Stressful working conditions<br>- Effort-Reward Imbalance, 10 items<br><br>- COPSOQ, emotional demands, short emotional scale 2 items	The items and response alternatives are published elsewhere:<br>(Siegrist J., 1996)<br>(Berthelsen, H. et al., 2013)	Imbalance between efforts and rewards were defined as an ERI ratio >1.<br>Emotional demands were defined as responding: "always" or "often" on both items.<br>Stress exposure strata was defined as having either ERI ratio >1 or emotional demands according to the definition given above.
Stress (used in non-response analysis)<br>"How have you felt during the last 3 months?<br>- I have had days where I constantly feel stressed."	- Not at all<br>- Sometimes<br>- Pretty often<br>- Almost all the time	Stress was defined as responding: "pretty often" or "almost all the time".

Working in preschool increases the risk of hearing-related symptoms - a cohort study among Swedish women, International Archives of Occupational and Environmental Health, Sofie Fredriksson, Jeong-Lim Kim, Kjell Torén, Lennart Magnusson, Kim Kähäri, Mia Söderberg, Kerstin Persson Waye; Department of Occupational and Environmental Medicine, Public Health & Community Medicine, Institute of Medicine, Sahlgrenska Academy, University of Gothenburg, sofie.fredriksson@gu.se

Online Resource 1 Continued

Questionnaire item in full text	Response alternatives	Derived variables
Employment status<br>"What is your current main employment status?"	- Currently working<br>- On leave of absence or on parental leave<br>- Job-seeker and/or in unemployment program<br>- Student<br>- Early retirement, disability pension or long-term sick leave (more than 3 months)<br>- Age retired<br>- Other	Currently working was defined as responding: "currently working".
Education level<br>"What is your highest attained education level?"<br><br>*Item answered by the population control cohort only.*	- Elementary school or similar<br>- 2-years upper secondary school<br>- 3–4 years upper secondary school<br>- University shorter than 3 years<br>- University 3 years or longer<br>- Not completed compulsory school	University- level education for controls were defined as responding: "university shorter than 3 years" or "university 3 years or longer".<br><br>All preschool teachers have a university degree.
Family monthly income<br>"How much is the household's approximate total monthly income before taxes?"	- 0–14999 SEK per month<br>- 15000–29999 SEK per month<br>- 30000–44999 SEK per month<br>- 45000–59999 SEK per month<br>- 60000 SEK or more per month	Family income of 30000 or more was used as a cut-off.
Education and income combined<br>Based on the variables "education level" and "family monthly income"		Three levels were defined:<br>- Both university education 3 years or longer and family income ≥30000 SEK<br>- Either university education 3 years or longer or family income ≥30000 SEK<br>- Lower education level than university and family income <30000 SEK
Smoking<br>"Have you ever smoked daily during at least a month?"	- No<br>- Yes	Smoking was defined as responding: "yes".
Family history of hearing loss<br>"Did any of your biological parents and/or siblings have a hearing loss before the age of 55 years?"	- No<br>- Yes<br>- Don't know	Family history of hearing loss was defined as responding: "yes".
Ear infections<br>"Have you ever had problems with otitis or ear infections?"	- Never or at most one time<br>- Yes, a few times<br>- Yes, many times and/or long-term	Recurrent infections was defined as responding: "yes, many times and/or long-term".
Tympanostomy tube<br>"Have you ever had ventilation tubes implanted in your eardrum?"	- No<br>- Yes	Tympanostomy tube was defined as responding: "yes".
Noisy activities leisure time<br>"How often in your leisure time do you participate in activities where noise or high sound levels occur?<br>With activity we mean for example: shooting/hunting, playing in a band, concert/disco, driving motorcycle, working with noisy tools/machines."	- Every day (4 points)<br>- Some or a few times each week (3 points)<br>- Some or a few times each month (2 points)<br>- Some or a few times each year (1 point)<br>- Never/rarely (0 points)	Leisure time exposure was defined as responding: "some or a few times a month" or more often.<br><br>Points calculated for sum score of leisure noise index are given in parenthesis for each response alternative.
Hearing protection leisure time<br>"Do you use hearing protection when you engage in noisy leisure activities?"	- Always/almost always (0 points)<br>- Often (1 point)<br>- Sometimes (2 points)<br>- Never/rarely (3 points)	Hearing protection in leisure time was defined as responding: "always/almost always" or "often".<br><br>Points calculated for sum score of leisure noise index are given in parenthesis for each response alternative.
Loud music in headphones<br>"How often do listen to portable music players with headphones?"<br><br>"When you listen, approximately how much of the maximum volume setting do listen to?"	- Every day (4 points)<br>- Some or a few times each week (3 points)<br>- Some or a few times each month (2 points)<br>- Some or a few times each year (1 point)<br>- Never/rarely (0 points)<br><br>- 25% of max volume (1 point)<br>- 50% of max volume (2 points)<br>- 75% of max volume (3 points)<br>- 100% of max volume (4 points)	Loud music in headphones was defined as responding: "some or a few times a month" in combination with a volume of "75% of max" or more.<br><br>Points calculated for sum score of leisure noise index are given in parenthesis for each response alternative.
