# Supplementary material for: Working in preschool increases the risk of hearing-related symptoms: a cohort study among Swedish women
Source: Int Arch Occup Environ Health. 2019 Jul 8;92(8):1179–90. doi: 10.1007/s00420-019-01453-0 (PMC6814644; doi:10.1007/s00420-019-01453-0)

Working in preschool increases the risk of hearing-related symptoms - a cohort study among Swedish women, International Archives of Occupational and Environmental Health, Sofie Fredriksson, Jeong-Lim Kim, Kjell Torén, Lennart Magnusson, Kim Kähäri, Mia Söderberg, Kerstin Persson Waye; Department of Occupational and Environmental Medicine, Public Health & Community Medicine, Institute of Medicine, Sahlgrenska Academy, University of Gothenburg, sofie.fredriksson@gu.se

Online Resource 2 Kaplan-Meier survival curves for hearing-related symptoms, stratified by recall.

The recall bias of retrospectively reported onset of hearing-related symptoms occurring between 24 and 65 years of age was assessed visually in Kaplan-Meier survival curves with events stratified by recall time (reporting onset 5, 10 or 15 years prior to the survey). Recall bias did not seem to affect the retrospective report of symptom onset differently in the two cohorts. The Kaplan-Meier survival curves were generally parallel within each recall strata for the two cohorts. However, preschool teachers generally had an earlier onset (steeper slope). Age of onset was not available for the symptom sound-induced auditory fatigue and therefore it is not included.

Hearing loss

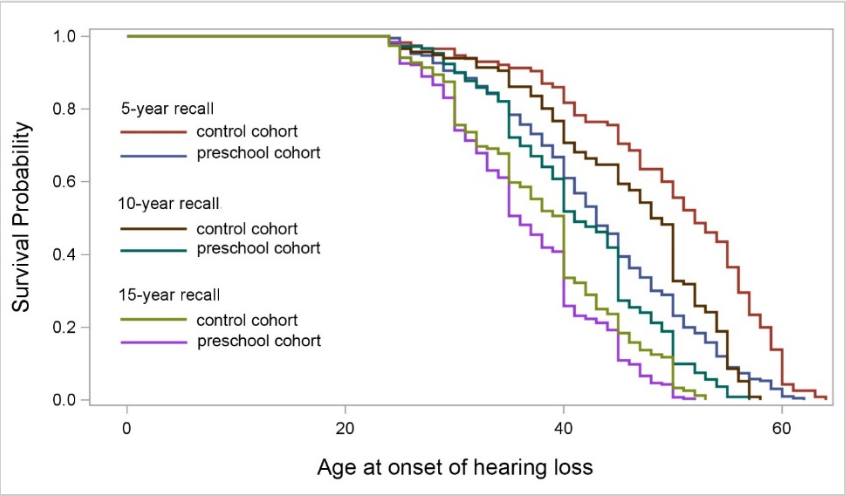

Tinnitus

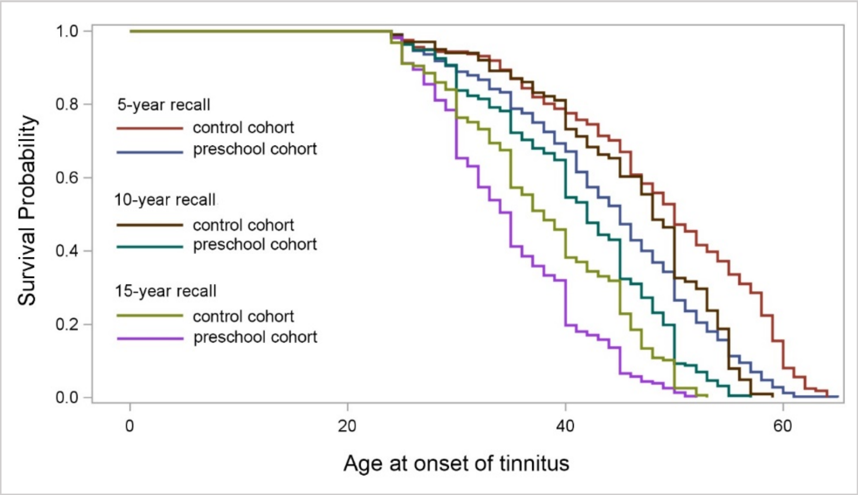

Online Resource 2 Continued

Difficulty perceiving speech

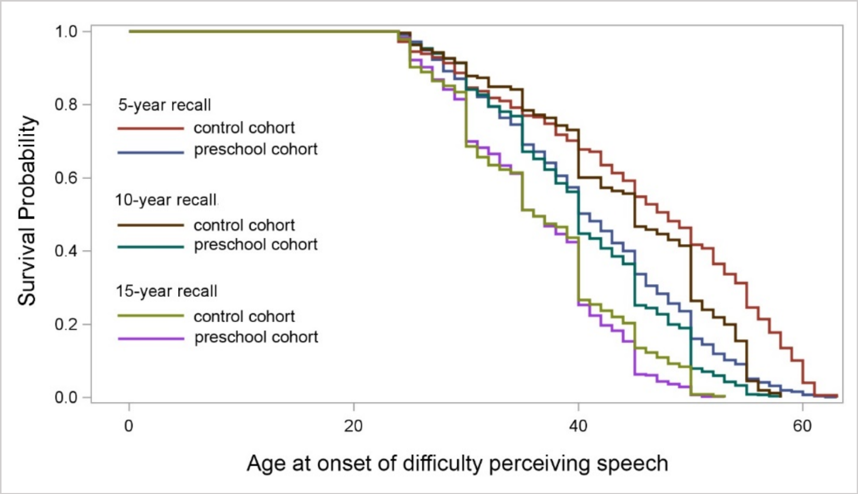

Hyperacusis

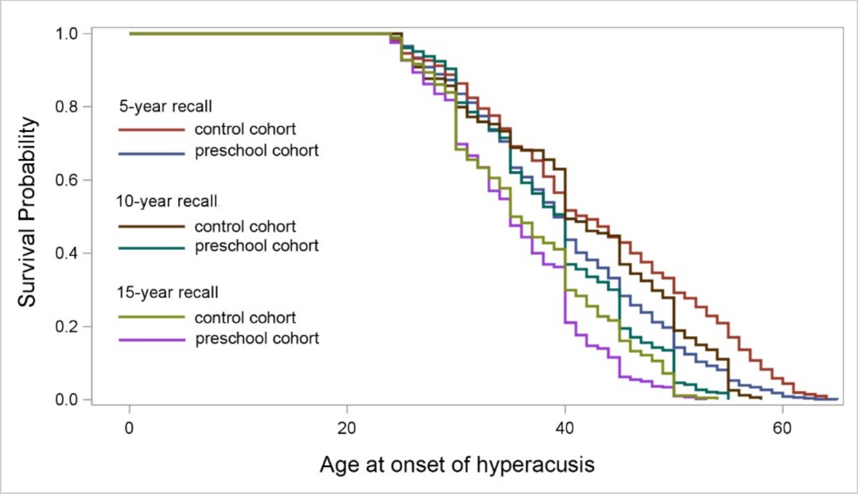

Supplement: Supplementary file 2 — Online Resource 2 shows the effect of recall due to retrospective reported symptom onset in Kaplan–Meier survival curves (PDF 364 kb) [file 420_2019_1453_MOESM2_ESM.pdf]
